# Total laparoscopic resection by medial-retroperitoneal approach using virtual navigation: two case reports of primary retroperitoneal schwannoma

**DOI:** 10.1186/s12957-021-02483-0

**Published:** 2022-01-04

**Authors:** Yuta Sato, Nobuhisa Matsuhashi, Yuto Sugie, Masashi Kuno, Shigeru Kiyama, Yoshihiro Tanaka, Naoki Okumura, Takao Takahashi, Takuya Saiki, Kazuhiro Yoshida

**Affiliations:** 1 Department of Gastroenterological Surgery, Pediatric Surgery, Gifu Graduate School of Medicine, Gifu, Japan; 2 Medical Education Development Center, Gifu University, Gifu, Japan

**Keywords:** Retroperitoneal schwannoma, Medial-retroperitoneal approach, Laparoscopic resection, Virtual navigation, Navigation surgery

## Abstract

**Background:**

We report two rare cases of retroperitoneal schwannoma completely resected by a laparoscopic medial-retroperitoneal approach aided by virtual navigation. Three-dimensional images have been used in liver and lung surgery, but there are few prior reports on retroperitoneal surgery.

**Case presentation:**

These two case reports are of a 60-year-old man and a 40-year-old man with asymptomatic retroperitoneal schwannoma. In both cases, the tumors were located in the right renal hilum and were close to the duodenum, right ureter, and inferior vena cava. Simulation using three-dimensional images was performed before surgery, and a medial-retroperitoneal approach was performed to secure a wide surgical field. During the operation, we confirmed the location of the main feeder and the relationship between the tumor and organs with those shown on the three-dimensional images and performed total laparoscopic resection.

**Conclusion:**

The medial-retroperitoneal approach provides operative safety. Preoperative simulation and intraoperative navigation with three-dimensional images, which can be freely rotated and interactively visualized from any angle, are useful methods to enhance the surgeon’s understanding of a patient’s specific anatomy and are especially effective when resecting a retroperitoneal tumor that is located in an anatomically deep and complex location.

## Background

Schwannomas are mostly benign tumors arising from Schwann cells of the peripheral and cranial nerves and are rarely situated in the retroperitoneal space [1]. We report two rare cases of asymptomatic retroperitoneal schwannoma that were found during regular medical checkups. In both cases, the tumors were located in the right renal hilum and were surrounded by the duodenum, right ureter, and inferior vena cava (IVC). We performed a simulation using a high-speed three-dimensional (3D) image analysis system (SYNAPSE VINCENT, Fuji Photo Film Co., Ltd., Tokyo, Japan) to obtain a more accurate localization of the anatomy of the tumors. In addition, we safely performed a total laparoscopic resection via a medial-retroperitoneal approach, the surgical technique for laparoscopic right colectomy on these two patients [2]. Preoperative simulation with virtual navigation is a useful method to share and understand the patient’s specific anatomy with surgeons from another department. Organs with segments, such as the liver and lungs, were suitable for the use of 3D images and have been used in a variety of ways [3–10], but it is also very effective for resecting a retroperitoneal tumor in a deep, anatomically complex location.

## Case presentation

Written informed consent was obtained from both patients for publication of the present case reports and the accompanying images.

### Case 1

**Fig. 1:**
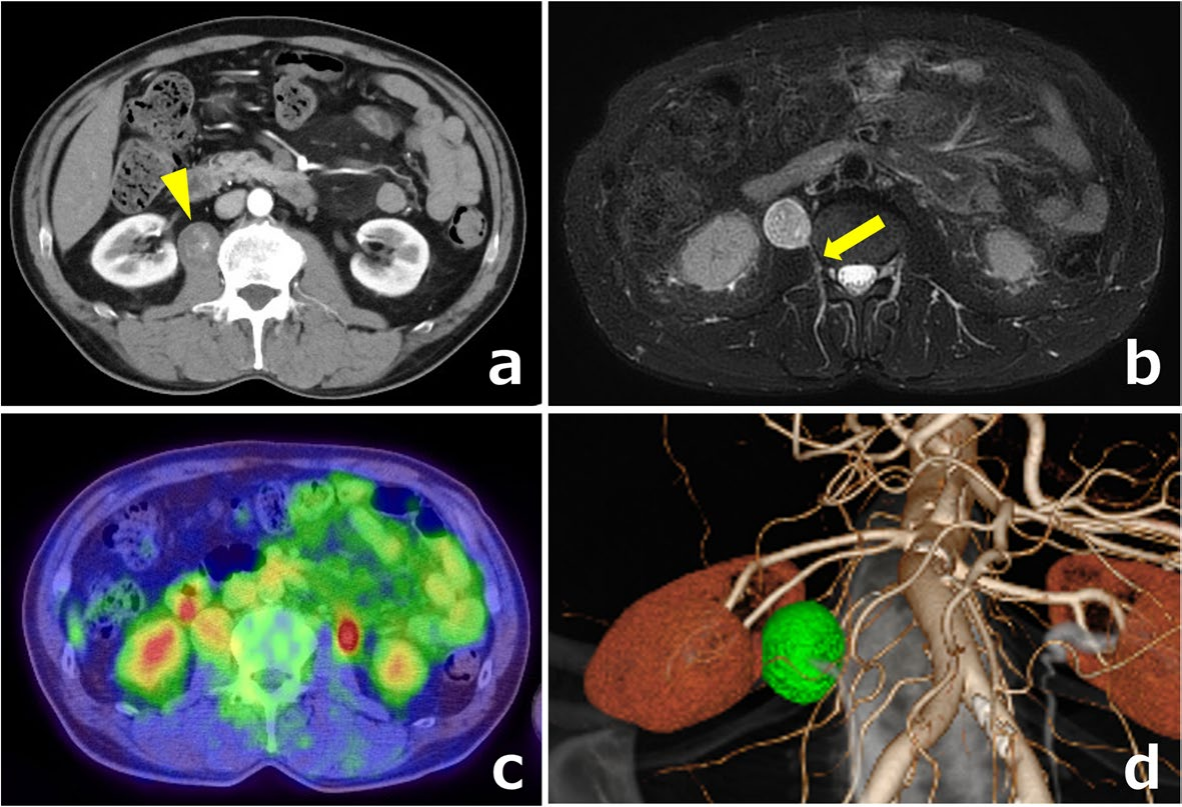
**a** CT showed a well-defined round 27 × 24 mm cystic mass (yellow arrowhead). **b** T2-weighted MRI showed a high-intensity lesion with the main feeder being a branch of the right third lumbar artery (yellow arrow). **c** PET-CT showed a mass with low FDG uptake. **d** The locations of the tumor and main feeder were identified before surgery on 3D images

**Fig. 2:**
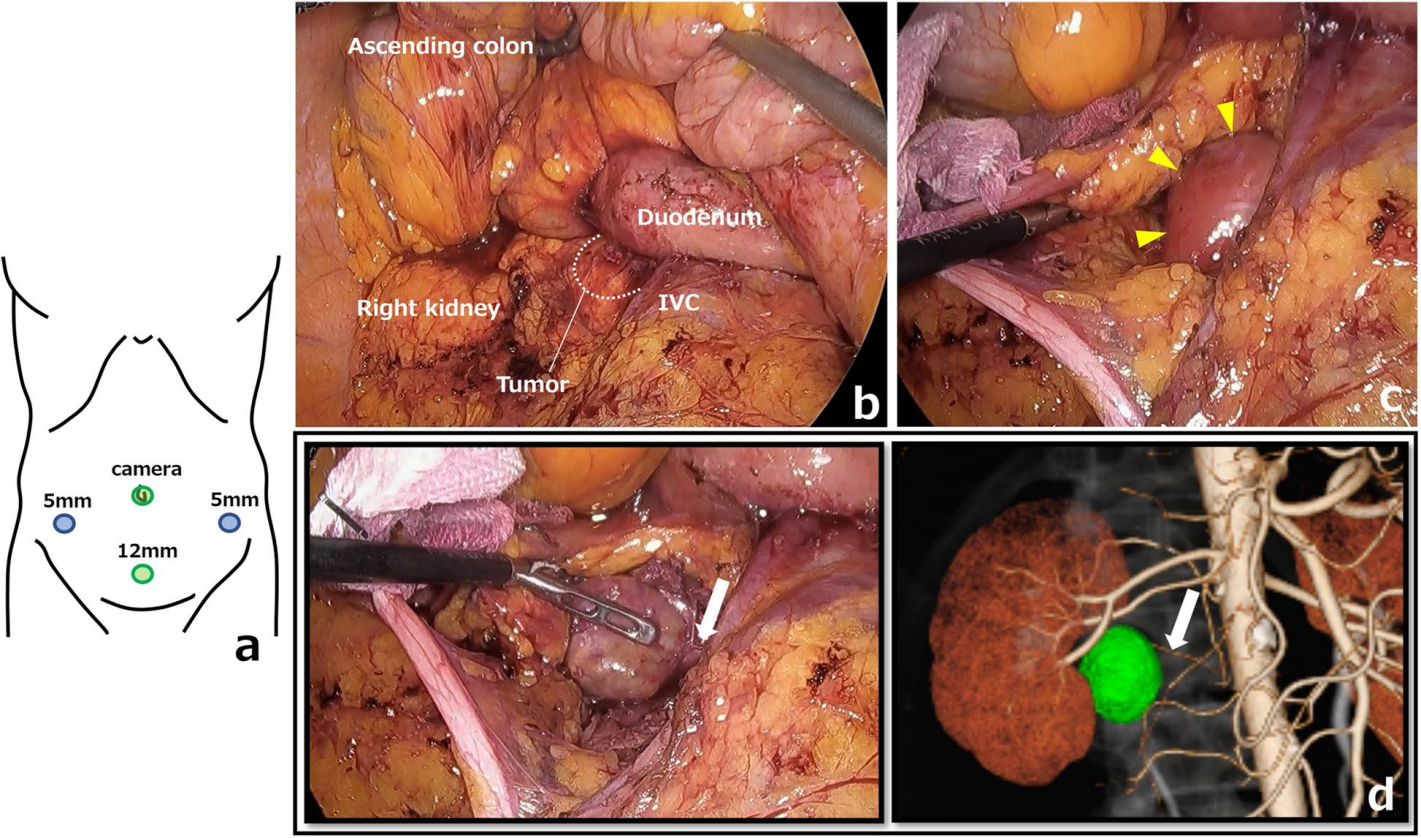
**a** Placement of the four ports. The assistant port was placed on the left side of the abdomen. **b** Laparoscopic view via the medial-retroperitoneal approach. **c** The tumor was in the right iliopsoas muscle (yellow arrowheads). **d** Comparison of the main feeder shown by 3D imaging and as seen in the operative view. The feeding artery (white arrow) depicted on the 3D images was consistent with that seen in the actual operative field

**Fig. 3:**
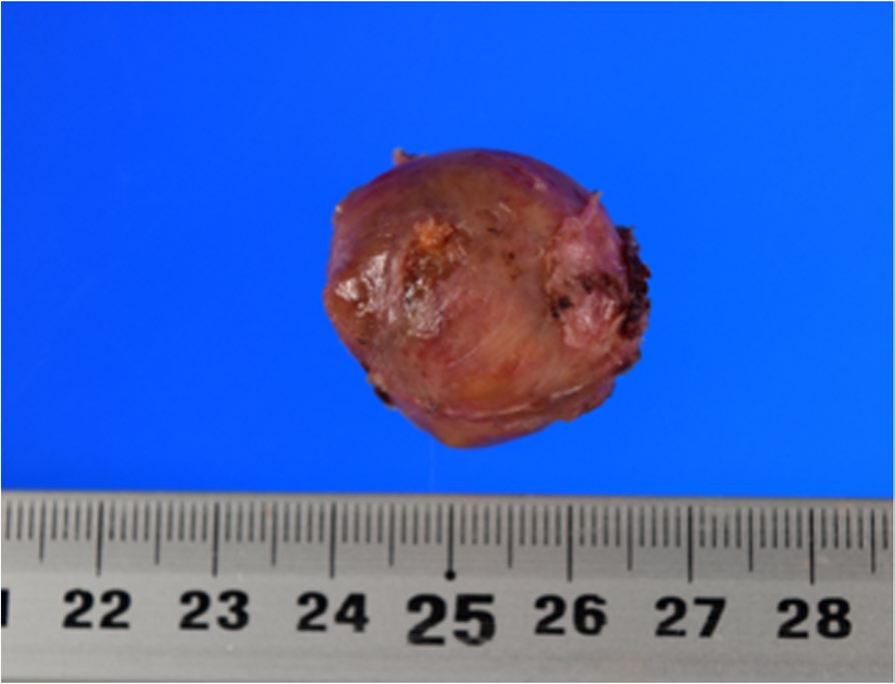
Surgical specimen

A 60-year-old man had a right retroperitoneal abdominal lesion that was found incidentally on abdominal computed tomography (CT) during a medical health checkup and was referred to our hospital for additional evaluation. Physical findings revealed a flat abdomen that was soft, not tender, and with no palpable mass. CT revealed a well-defined round 27 × 24 mm cystic mass in the right retroperitoneum (Fig. 1a). The mass was located in the right renal hilum, dorsal to the IVC, and ventral to the right iliopsoas muscle. T2-weighted magnetic resonance imaging (MRI) showed a high-intensity lesion, with the main feeder being a branch of the right third lumbar artery (Fig. 1b). A mass showing low fluorodeoxyglucose (FDG) uptake (maximum standardized uptake value [SUV] 3.74) was observed on positron emission tomography and computed tomography (PET-CT) in the same location (Fig. 1c). A tentative preoperative diagnosis of malignant tumor of undermined origin, schwannoma, or paraganglioma was considered based on these radical findings. Since the patient was taking two antiplatelet medications, we decided not to perform a biopsy, but to perform surgery as a diagnostic treatment. We used the SYNAPSE VINCENT to convert Digital Imaging and Communication in Medicine (DICOM) data of the contrast-enhanced CT images to 3D images, and the locations of the lesion and main feeder were clearly identified before surgery (Fig. 1d). We performed laparoscopic resection through four ports after inducing pneumoperitoneum (Fig. 2a) and a medial-retroperitoneal approach to provide a favorable surgical field (Fig. 2b). After incision of the retroperitoneum, the tumor was observed in the right iliopsoas muscle (Fig. 2c). After taping the right ureter, the right iliopsoas muscle was split to expose the tumor, and the feeding artery from the lumbar artery was visually recognized. We synchronized the surgical image and the 3D image on the monitor side-by-side for virtual navigation and located the feeding artery that led to the dorsal side of the tumor (Fig. 2d). The tumor specimen was removed via the umbilical wound (Fig. 3). The operative time was 116 min, and the operative blood loss was approximately 3 mL. A pathological examination of the tumor confirmed the diagnosis of ancient schwannoma with clear surgical margins. The patient recovered without complications and was discharged on the 6th day after surgery.

### Case 2

**Fig. 4:**
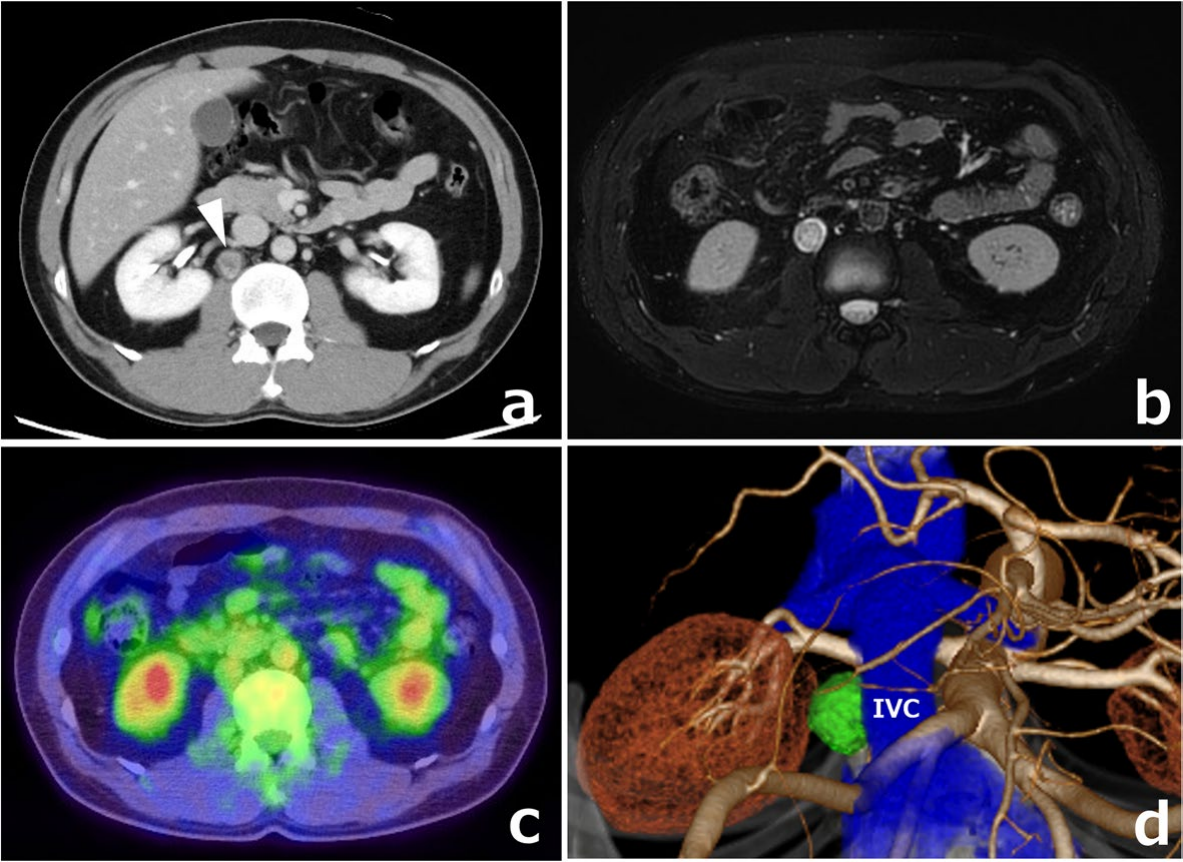
**a** CT showed a tumor (white arrowhead) on the dorsal side of the IVC. **b** T2-weighted MRI showed a high-intensity lesion. **c** A mass with low FDG uptake was observed on PET-CT. **d** SYNAPSE VINCENT was used to convert DICOM data of the CT images to 3D images

**Fig. 5:**
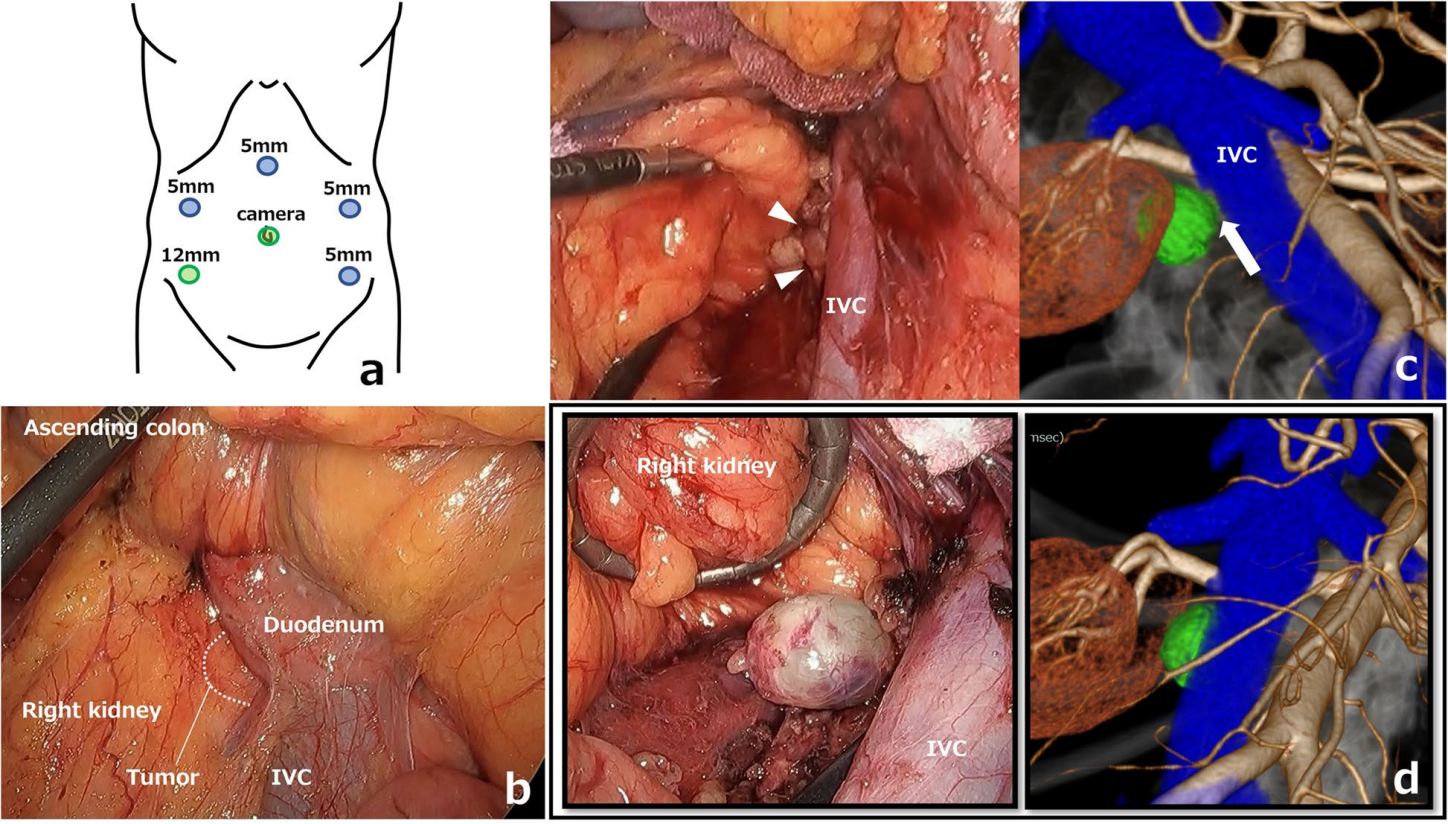
**a** Placement of the six ports. The primary surgeon used the two ports on the right side, while the assistant used the three ports on the left side and in the cardiac fossa (added intraoperatively). **b** Laparoscopic view via the medial-retroperitoneal approach. **c** Comparison of the tumor shown by 3D imaging and as seen in the operative view. The tumor was on the dorsal side of the IVC (white arrowheads), but no invasion was shown on the 3D image (white arrow). **d** Comparison of the 3D image and operative view

**Fig. 6:**
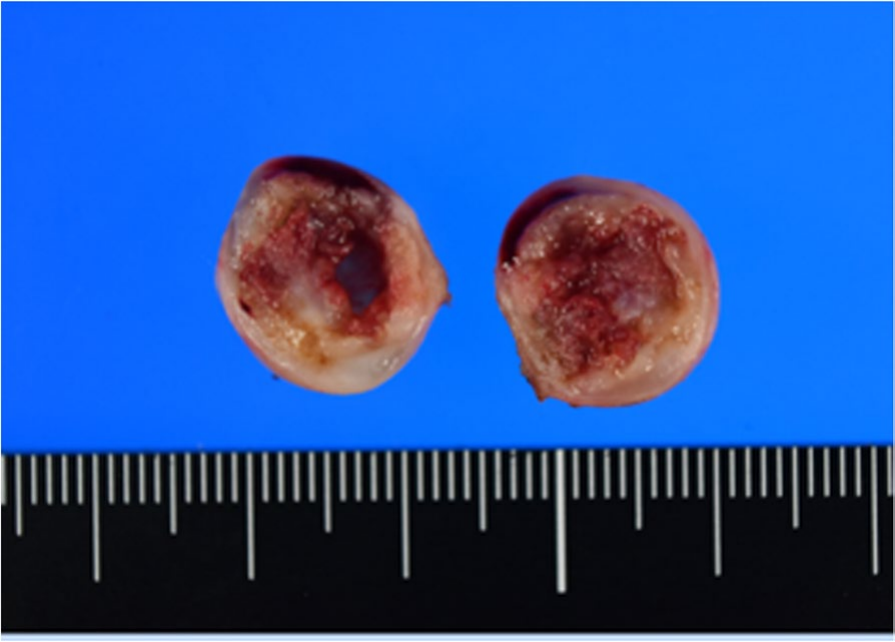
Surgical specimens

In a 40-year-old man with rheumatoid arthritis, a retroperitoneal lesion was incidentally found on a screening CT. His general physical and abdominal examinations revealed normal findings. CT revealed a solid tumor of 18 × 17 mm on the dorsal side of the IVC and the right renal vein (Fig. 4a). T2-weighted MRI showed a high-intensity lesion (Fig. 4b), and a mass with low FDG uptake (max SUV 2.54) was observed on PET-CT (Fig. 4c). Biopsy was deemed difficult due to the location and size of the tumor, and surgery was planned for diagnostic and therapeutic purposes. In the preoperative conference, we discussed the patient’s 3D images with a urologist and performed laparoscopic surgery after fully understanding the location of the tumor (Fig. 4d). We performed a medial-retroperitoneal approach through six ports (Fig. 5a, b). An incision in the retroperitoneum and taping of the right ureter revealed the tumor, which was in contact with the dorsal side of the IVC, but when the 3D images were rechecked intraoperatively, there was no invasion and the dissection could be performed safely (Fig. 5c). During the surgery, this 3D image was displayed on two monitors side by side with the laparoscopic image and could be freely rotated and viewed from any angle as the surgery progressed (Fig. 5d). The use of intraoperative virtual navigation was effective in identifying the tumor deep within the retroperitoneum. The cut surface of the tumor showed red-brownish cyst formation and a yellowish-white rim (Fig. 6). The operative time was 147 min, and the operative blood loss was approximately 5 mL. He was discharged on the 7th day after surgery.

## Discussion and conclusions

Retroperitoneal schwannomas are rare, accounting for just 0.5–3.0% of all schwannomas and only 1% of all peritoneal neoplasias [11]. As these tumors are usually asymptomatic and discovered by chance or in the course of the evaluation of an unrelated health problem, they may cause a delay in their early diagnosis and treatment. The ideal treatment for schwannoma is complete resection of the tumor and capsule without injuring the adherent organs [12].

**Fig. 7:**
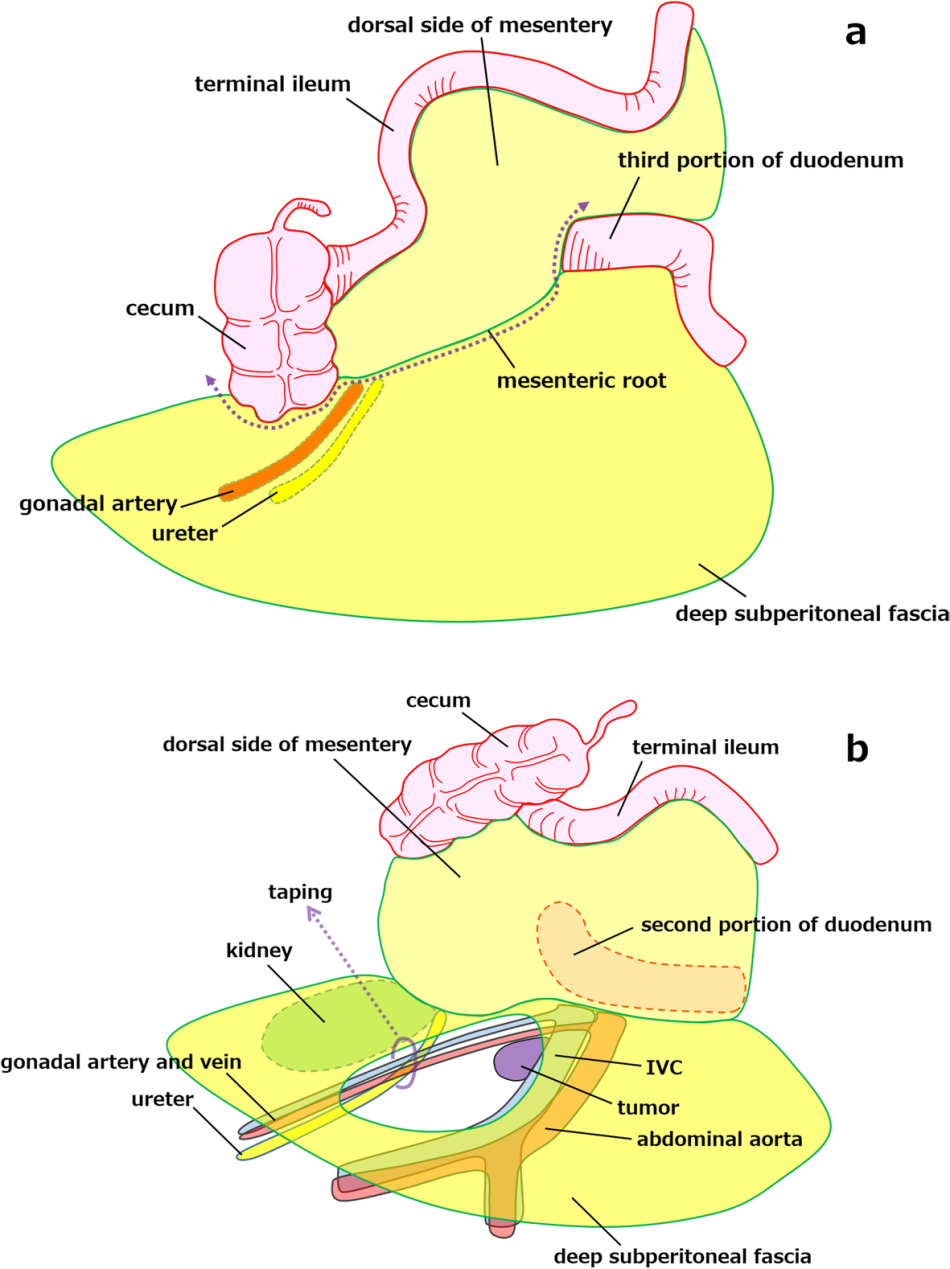
**a** The medial-retroperitoneal approach begins with an incision of the peritoneum at the mesenteric root (dotted arrow), after which the dissection plane can be entered to the ventral side of the deep subperitoneal fascia. **b** This approach involves wide dissection of the ventral side of the deep subperitoneal fascia and provides a favorable surgical field

With recent advances in the field of minimally invasive surgery, several laparoscopic approaches to retroperitoneal schwannomas have been reported [13, 14]. Laparoscopic surgery, which has become a useful and feasible option for this procedure, is associated with minimal invasiveness and early postoperative recovery. The medial-retroperitoneal approach is the surgical technique for laparoscopic right colectomy [2]. The anatomical landmarks of the medial-retroperitoneal approach are the third portion of the duodenum, the mesenteric root of the terminal ileum, and the caudal portion of the cecum. This approach begins with an incision of the peritoneum at the base of the mesentery of the intestine. After lifting the cecum or the terminal ileum ventrally, the dissection is performed just dorsal to the right fusion fascia of Toldt and sufficiently to the cranial side and then continues medially to the second portion of the duodenum (Fig. 7a). We decided to perform this approach based on simulating the surgery using the 3D images obtained preoperatively. The medial-retroperitoneal approach is the preferred procedure in laparoscopic resection of retroperitoneal tumors because it involves wide dissection of the ventral side of the deep subperitoneal fascia and the surface of the duodenum and thus provides a favorable surgical field (Fig. 7b).

A retroperitoneal tumor can be in close contact with structures such as the duodenum, right renal vein, and IVC, which often require meticulous dissection to avoid damage. Thus, precise knowledge of the surgical anatomy of each patient is absolutely mandatory to ensure a safe operation. Since the SYNAPSE VINCENT can create 3D images based on preoperative CT images, so there is no burden on the patients and making it easier for the surgeon to visually recognize the anatomy and have a common understanding. In the fields of hepatic [3, 4] and thoracic surgery [5, 6] in particular, preoperative simulation has been widely used, and the safety and efficacy of 3D images for preoperative assessment have already been described [7–10]. The use of 3D images for retroperitoneal tumor resection, as in our case, is a very effective and new technique with few prior studies.

Preoperative simulation and intraoperative navigation with 3D images are useful methods to enhance the surgeon’s understanding of a patient’s specific anatomy and are especially effective when resecting a retroperitoneal tumor in an anatomically deep and complex location. Three-dimensional images can be rotated freely and visualized interactively from any angle to provide an overview of the 3D relationships of the retroperitoneal organs. In addition, information about the location of the tumor can be shared between each operator and doctors from other departments, ensuring secure resection in a narrow retroperitoneal space near important densely packed organs. This imaging technique not only is helpful for laparoscopic retroperitoneal procedures but is an excellent tool for the education of surgical trainees and for medical students studying surgical anatomy.

## Data Availability

Not applicable

## Abbreviations

CT: Computed tomography
DICOM: Digital Imaging and Communication in Medicine
FDG: Fluorodeoxyglucose
IVC: Inferior vena cava
MRI: Magnetic resonance imaging
PET-CT: Positron emission tomography and computed tomography
SUV: Standardized uptake value
3D: Three-dimensional
